# LncRNA-Encoded Peptide: Functions and Predicting Methods

**DOI:** 10.3389/fonc.2020.622294

**Published:** 2021-01-14

**Authors:** Jiani Xing, Haizhou Liu, Wei Jiang, Lihong Wang

**Affiliations:** ^1^ Department of Pathophysiology, Medical College of Southeast University, Nanjing, China; ^2^ Department of Biomedical Engineering, Nanjing University of Aeronautics and Astronautics, Nanjing, China; ^3^ Jiangsu Provincial Key Laboratory of Critical Care Medicine, Nanjing, China

**Keywords:** long non-coding RNA, peptide, translation, cancer, Ribo-seq

## Abstract

Long non-coding RNA (lncRNA) was originally defined as the representative of the non-coding RNAs and unable to encode. However, recent reports suggest that some lncRNAs actually contain open reading frames that encode peptides. These coding products play important roles in the pathogenesis of many diseases. Here, we summarize the regulatory pathways of mammalian lncRNA-encoded peptides in influencing muscle function, mRNA stability, gene expression, and so on. We also address the promoting and inhibiting functions of the peptides in different cancers and other diseases. Then we introduce the computational predicting methods and data resources to predict the coding ability of lncRNA. The intention of this review is to provide references for further coding research and contribute to reveal the potential prospects for targeted tumor therapy.

## Highlights

- We summarize the mechanisms of mammalian lncRNA-encoded peptides in regulating biological processes.
- We review the functions of lncRNA-encoded peptides in human diseases, especially in cancers.
- We sum up the classic computational methods and data resources for predicting lncRNA-encoded peptides.
- This review may lay the foundation for further coding research and reveal the potential prospects for targeted tumor therapy.

## Introduction

Non-coding RNA (ncRNA) is widely described as a class of RNA molecules transcribed from genomic DNA without coding capability (1). But with the improvements of bioinformatics and high-throughput technologies, open reading frames (ORFs) have been found in ncRNAs, such as pri-miRNA, circular RNA (circRNA), and long non-coding RNA (lncRNA) (2). These discoveries suggest that ncRNAs may encode proteins or peptides. The ORF of pri-miRNAs can encode peptides if they are directly transported to the cytosol without processing. For example, miPEP171b and miPEP165a, two peptides respectively encoded by pri-miR171b in *Medicago truncatula* and the pri-miR165a in *Arabidopsis thaliana*, can regulate root development by reducing expression of target genes (3). Besides ORFs, circRNA can be translated into proteins *via* internal ribosome entry site (IRES)-driven or N^6^-methyladenosine (m^6^A)-mediated initiation (4). Recently, a new coding type is reported in circRNA SHPRH which generates a protein from genetic codes overlapping. This protein protects itself from degradation by ubiquitination in gliomas (5, 6). Moreover, it has been found that a special circRNA is constituted by a head-to-tail circle form of lncRNA LINC-PINT exon 2, and encodes a peptide PINT87aa (7).

In addition to these ncRNAs, the regulatory mechanisms and functions of lncRNA coding products receive much concerns recently. LncRNAs are transcripts longer than 200nt and mainly located in the nucleus. The number of lncRNAs may exceed that of protein coding transcripts but its expression is generally lower. Owing to its abnormal expression and mutation, lncRNA is involved in many diseases, especially in cancers (8–10). Recent studies indicate that the translation of lncRNAs is driven by ORF, and some lncRNAs exert their functions through their coding peptides (2). For example, the 53-amino acid (aa) peptide encoded by lncRNA HOXB-AS3 can inhibit colon cancer (11). LINC00689 encodes a 50aa peptide, which is highly similar to the signal recognition particle 19 kDa protein (SRP19), and this coding ability was accelerated by the phosphorylated translation initiation factor eIF4E (12). Even though, it is rather difficult to evaluate the coding capacity of lncRNA due to high similarity to mRNA in structure, and sometimes the coding products are encoded in introns or overlapping exons of different genes. Only a minority of lncRNAs are functionally annotated at present, and there are still large amount of lncRNA-encoded products need to be discovered (13).

The coding products of lncRNAs has been studied in plants and invertebrates for a long time (14). As early as 2002, a soybean research discovered that a 679nt lncRNA, translated by early nodulin 40 gene, encoded two small peptides interacting with sucrose synthase (15). In addition, the lncRNA Toddler in zebrafish encodes a 58aa peptide and promoted gastrulation movements (16). But the study of lncRNA-encoded peptides in mammals is just beginning. Even though, there are still many exciting results achieved. Hitherto, some articles have introduced peptides encoded by lncRNAs in a wide range of species, and most of them are peptide-oriented (11, 17–19). However, in this review, we focused on the functions of mammalian lncRNA-encoded peptides oriented toward pathways and diseases, especially in tumors. We also included representative computational approaches and data resources for predicting the coding possibility.

### The Pathways Regulated by Long Non-Coding RNA-Encoded Peptides

#### Inhibiting Mammalian Target of Rapamycin Complex 1

The lysosome has the ability to degrade and recycle macromolecules, and its acidification is regulated by v-ATPase and the mammalian target of rapamycin complex 1 (mTORC1). V-ATPase activates mTORC1 and improves the excretion of essential amino acids from lysosomes (20, 21). LncRNA LINC00961 contains three ORFs, one of which has been confirmed to encode the polypeptide SPAR by tandem mass spectrometry (MS). By specifically blocking v-ATPase, SPAR prevents mTORC1 activation that is stimulated by amino acids. Matsumoto et al. found that cell proliferation and muscle regeneration were accelerated when they knocked out the SPAR polypeptide in mice and injected a toxin into the muscle to induce injury (22), which suggested that SPAR was involved in the process of mTORC1 influencing muscle function (23). Besides, Spencer HL et al. found both LINC00961 and its coding peptide had independent function. LINC00961 itself acts as an angiogenesis inhibitor and interacts with actin-binding protein Tβ4 while its peptide promotes angiogenesis and binds to actin-binding protein SYNE1 (24). Moreover, a micropeptide LEMP encoded by lncRNA MyolncR4 also functions on muscles. It is shown that muscle development defect manifested in the reduction of muscle size and weight in LEMP KO mice. LEMP also promotes skeletal muscle activation and new fibers formation (25).

#### Regulating Sarcoplasmic Reticulum Ca^2+^-ATPase

The sarcoplasmic reticulum Ca^2+^-ATPase (SERCA) mediates muscle relaxation by pumping Ca^2+^ back into the sarcoplasmic reticulum, and its activity can be inhibited by sarcolipin (SLN) and phospholamban (PLN) (26). Some lncRNA-translated peptides have been reported as the regulators of SERCA. Skeletal muscle-specific LINC00948 generates a micropeptide designated as myoregulin (MLN), which can form a transmembrane alpha-helix. In addition, MLN has a similar hydrophobic binding motif as SLN and PLN for inhibiting the SERCA pump activity (27). In contrast, LOC100507537 encodes a 34aa peptide DWORF, which activates the SERCA pump. This DWORF peptide can counteract the SERCA inhibitor and reduce muscle contraction time (28). Furthermore, one lncRNA with two functional ORFs in Drosophila has been renamed as sarcolamban, which may have homology with SLN and PLN in vertebrates, as revealed through a cross-species sequence analysis (29).

#### Participating in Messenger RNA Decay

Non-sense-mediated mRNA decay (NMD) is the mRNA monitoring mechanism in eukaryotes, which decomposes mRNAs with premature termination codons. These mRNAs are produced during gene expression or cellular homeostasis maintenance processes (30). LINC01420, which is expressed only in mammalian cell lines, produces a polypeptide called NoBody. NoBody has neither homologous proteins nor a secondary structure, so its function can be measured only by proteomic techniques. In addition, the results suggest that NoBody is involved in mRNA decapping and degradation, which causes a decrease in cytoplasmic processing bodies (P-bodies) and a reduction in cellular NMD substrates (31). Recently, some studies have discovered that the NMD process may occur during the interaction between lncRNAs and ribosomes (32, 33). Another lncRNA, EPR, on the polyribosome is identified to have a 213 nucleotide-long ORF, which can recover mRNA stability by weakening the effect of the KH-type splicing regulatory protein (KHSRP), while overexpressing the EPR-encoded polypeptide can promote epithelial cell tight junctions (34).

#### Stimulating Mitochondria

The mitochondrial inner membrane, which is the membrane unit wrapped around the mitochondrial matrix, contains a large quantity of cardiolipin and proteins. This membrane is responsible for metabolite transport, oxidative phosphorylation, ATP synthesis, and mitochondrial fission and fusion (35). Stein et al. found that LINC00116 could encode a 56aa microprotein called mitoregulin (Mtln), which localized to the mitochondrial inner membrane. Because Mtln forms a high molecular weight complex *via* the sticky intrinsically disordered protein regions (IDPR), it can strengthen the effect of the mitochondrial respiratory chain (MRC) and reactive oxygen species (36). Makarewich et al. demonstrated that the function of 1510011k16Rik in mice was the same as its homolog LINC00116 in humans. In addition, 1510011k16Rik produces the micropeptide regulator of β-oxidation (MOXI). MOXI interacted with mitochondrial trifunctional protein to meet the energy requirements of an increasing metabolism and to control biological activity in other latent pathways (37). This discovery was subsequently confirmed by Chugunova et al. These authors suggested that MOXI can affect the activity of cytochrome b5 reductase 3 (Cyb5r3) associated with lipid metabolism and stimulate the MRC complex I (38).

**Figure 1:**
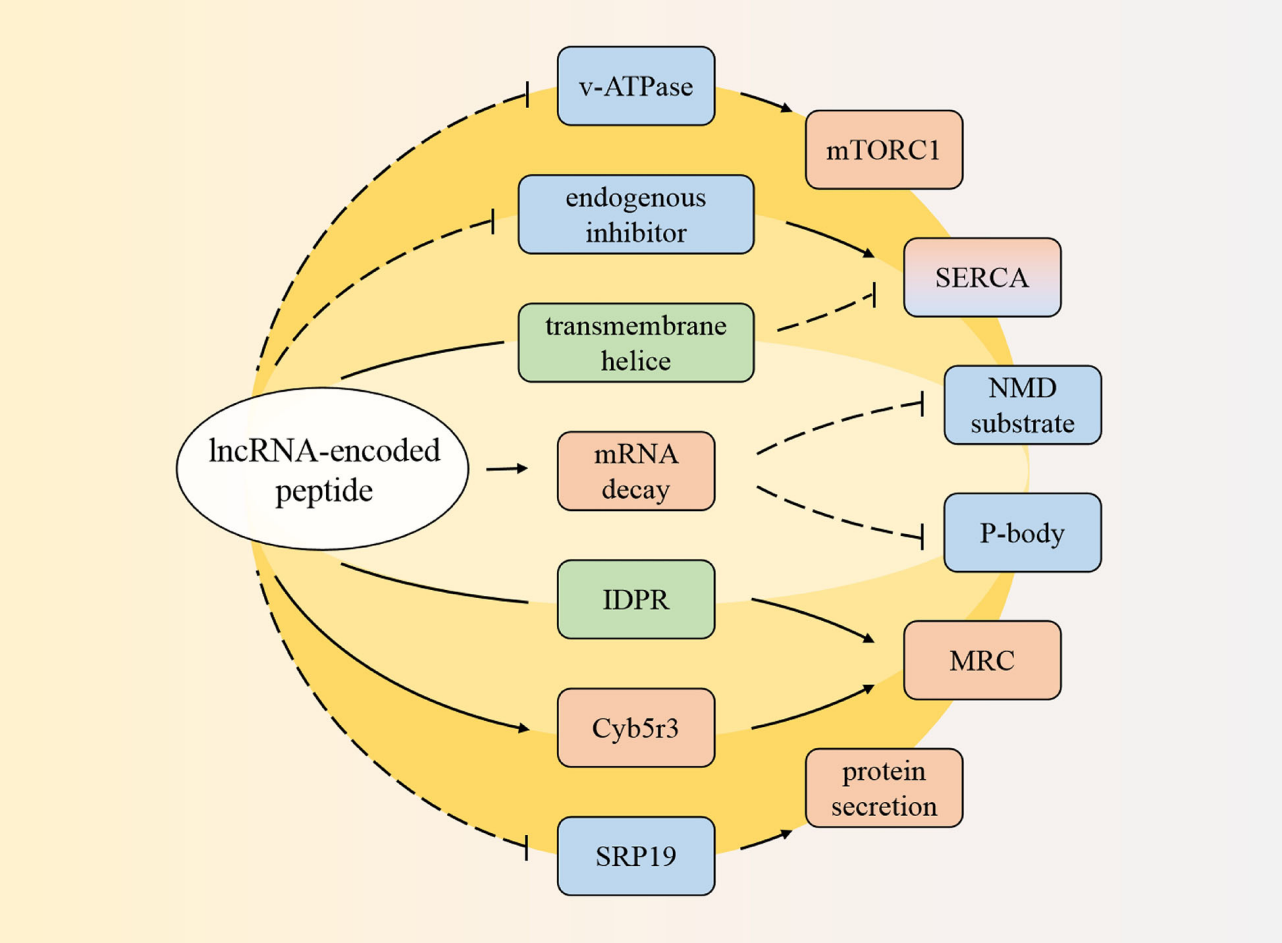
Pathways regulated by the coding peptides. The peptide can prevent mammalian target of rapamycin complex 1 (mTORC1), and regulate sarcoplasmic reticulum Ca^2+^-ATPase (SERCA). One of them can also involve in messenger RNA (mRNA) decay to decrease cytoplasmic processing bodies (P-body) and cellular nonsense-mediated mRNA decay (NMD) substrate. In addition, one coding product has intrinsically disordered protein regions (IDPR), affects cytochrome b5 reductase 3 (Cyb5r3) to stimulate mitochondrial respiratory chain (MRC). Another coding peptide interferes signal recognition particle 19kDa protein (SRP19). Red: promotion; blue: inhibition; green: structure.

In summary, lncRNA-encoded peptides can prevent mTORC1 activation to influence muscle function and regulate the SERCA pump activity to control muscle contraction time. Additionally, they can not only involve in mRNA stability and then mediate gene expression or cellular homeostasis maintenance processes, but also strengthen the MRC effect and ROS production. All above these were shown in **Figure 1** and **Table 1**.

**Table 1:** The function of long non-coding RNA (lncRNA)-encoded peptides in mammals.

LncRNA	Coding peptide	Length	Function of coding product	Reference
LINC00689	STORM	50aa	Inhibit protein secretion.	(12)
LINC00961/5430416O09Rik (mouse)	SPAR	90aa	Inhibit mTORC1.	(22)
	SPAAR		Promote muscle development.	(24)
MyolncR4	LEMP	56aa	Promote muscle development.	(25)
LINC00948/2310015B20Rik (mouse)	MLN	46aa	Inhibit SERCA.	(27)
NONMMUG026737 (mouse)	DWORF	34aa	Activate SERCA.	(28)
LINC01420	NoBody	63aa	Decrease P-bodies and mRNA.	(31)
EPR (mouse)	EPRp	71aa	Promote epithelial tight junction.	(34)
LINC00116/1500011k16Rik (mouse)	Mtln	56aa	Promote mitochondrion.	(36–38)
	MOXI (mouse)			
HOXB-AS3	–	53aa	Suppress colon cancer.	(39)
LOC90024	SRSP	130aa	Promote colon cancer.	(40)
LINC00266-1	RBRP	71aa	Promote colon cancer.	(41)
LINC00998	SMIM30	59aa	Promote liver cancer.	(42)
HBVPTPAP	HBVPTPAP	145aa	Suppress liver cancer.	(43)
LINC00278	YY1BM	21aa	Suppress esophageal cancer.	(44)
LINC00665	CIP2A-BP	52aa	Suppress breast cancer.	(45)
LINC00908	ASRPS	60aa	Suppress breast cancer.	(46)
meloe	MELOE-3	54aa	Possess immune tolerance.	(47)
MIR155HG	P155	17aa	Suppress autoimmune inflammation.	(48)
Aw112010	–	84aa	Cause immune response.	(49)

### The Functions of Long Non-Coding RNA-Encoded Peptides in Cancer

Lately, peptides as tumor biomarkers have attracted increasing attention in clinical cancer treatments. Chakraborty et al. identified that the lncRNA translation products had a similar expression in 11 carcinoma cell lines, which showed great stability and succeeded as a general biomarker for cancer (50). Moreover, the coding peptides in other ncRNAs also function in malignant tumors. For example, a knockdown of microprotein CASIMO1, encoded by a ncRNA, can affect breast cancer cell proliferation. CircPPP1R12A encodes a 73aa protein, which helps colon cancer to metastasize (6, 51). Therefore, the lncRNA-encoded products are expected to be promising cancer targets and biomarkers in tumor therapies (18).

#### Colon Cancer

The peptide encoded by lncRNA HOXB-AS3 is involved in metabolic reprogramming to inhibit the growth of colon cancer. The ORF of HOXB-AS3 encodes a 53aa endogenous peptide. Kaplan-Meier analysis suggests that a lower expression of this 53aa peptide is associated with a shortened survival time in colon cancer patients. To distinguish whether the lncRNA or its peptide produced a degenerative effect in colon cancer, researchers mutated the start codon to delete the coding function of HOXB-AS3 and found that it was the coding peptide that influenced the formation of cancer cell colonies. This peptide decreases pyruvate kinase formation and reduces lactic acid production to inhibit colon cancer cell proliferation by antagonizing the splicing factor hnRNP A1 (39). Interestingly, the difference is that lncRNA HOXB-AS3 is downregulated in colon cancer but upregulated in acute myeloid leukemia. Papaioannou et al. deemed that the overlap of the lncRNA HOXB-AS3 transcript variants was limited in the two diseases. HOXB-AS3, which promotes the proliferation of acute white myeloid cells, is mainly located in the nucleus. It has a low correlation with polyribosome fragments, and there is no ribosomal protein enrichment in the eluate (52).

After confirming the anticancer activity of the lncRNA HOXB-AS3-encoded peptide, Yan et al. recently provided other verification on a 130aa protein translated by lncRNA LOC90024 in advanced colorectal cancer. This 130aa-protein was named splicing regulatory small protein (SRSP), due to its interaction with serine- and arginine-rich splicing factor 3 (SRSF3) to regulate mRNA splicing. The formation of the transcription factor long Sp4 isoform contains an adequate transactivating domain induced by SRSP binding to SRSF3 and regulates oncogene expression. Consequently, SRSF can promote colon cancer cell proliferation, migration, and invasion. In this example, alternative splicing provides an additional regulatory mechanism for transcription factor activity in cancer and leads to the activation of oncogenes and tumor progression (40).

Meanwhile, Yan et al. found 55 lncRNAs with different expression and coding potentials in the SW480 and SW620 cell lines and finally confirmed that among these, LINC00266-1 was able to produce RNA-binding regulatory peptide (RBRP). The N^6^-methyladenosine (m^6^A) reader mediates m^6^A recognition on RNAs, such as c-Myc mRNA. Thereby, RBRP, which binds to the m^6^A reader IGF2BP1, can strengthen mRNA stability and increase the incidence of tumors. This m^6^A recognition abnormality reveals a new way in which to target cancer (41).

#### Liver Cancer

An endogenous peptide SMIM30 has positive effect during the progression of hepatocellular carcinoma. Ribosomal protein S6 (RPS6) is the point to identify ORF existence. After being evaluated by software-coding potential calculator, LINC00998 sticks out from other RPS6-related lncRNAs and has high coding potential score. The study discovered that LINC00998 can produce a 7.3 kDa peptide SMIM30 on cell membrane. If mutate the start codons of LINC00998’s ORF, SMIM30 cannot perform its carcinogenesis by activating protein tyroaine kinase membrane anchoring and MAPK pathway (42).

It has been identified that lncRNA HBVPTPAP is overexpressed in the cytoplasm of HepG2 cell and inhibits cell proliferation significantly. There are two potential coding regions in HBVPTPAP, but one of them is excluded due to an overlap with the reference gene’s exon. Another region can produce a polypeptide which interacts paired immunoglobulin like type 2 receptor alpha intracellular domain to activate JAK/STAT signaling pathway. Meanwhile, the peptide can also increase mitochondrial membrane potential and early apoptosis rate (43).

#### Male Esophageal Squamous Cell Carcinoma

Yin Yang 1-binding micropeptide (YY1BM) is a potential anticancer factor and can be encoded by the ORF in the first exon of LINC00278. The demethylated m^6^A can improve this translation efficiency, which is mediated by the ALKBH5 protein induced by cigarette smoking. YY1BM can affect the binding between the transcription factor Yin Yang 1 and the androgen receptor to inhibit the transcription of eukaryotic elongation factor 2 kinase, which is the key factor in tumor adaptation to nutrient deprivation. A YY1BM knockout can reverse the nutrient deprivation-induced apoptosis in esophageal squamous carcinoma cells (44).

#### Breast Cancer

In triple-negative breast cancer (TNBC), a micropeptide CIP2A-BP translated from LINC00665 is found to inhibit the metastasis to lung. When Smad signal transduction pathway is stimulated by TGF-β and then lead to the increasing combination between translation inhibitory protein 4E-BP1 and eIF4E, LINC00665’s translation is suppressed and its coding peptide is reduced. Downregulated CIP2A-BP induces low activity of cancerous inhibitor of PP2A and promotes cancer metastasis and invasion in mouse mammary tumor model. While CIP2A-BP overexpression inhibits AKT phosphorylation and the TNBC progression (45).

At the same time, another peptide ASRPS produced by the third ORF of LINC00908 can inhibits TNBC. It is found that LINC00908 transcription is positively correlated with ERα and regulated directly by it, which cause the expression of ASRPS is low in TNBC. ASRPS can enhance the interaction between signal transducer and activator of transcription 3 and vascular endothelial growth factor thus blocks the blood vessel formation (46).

LncRNA EPR regulates cell proliferation and levels of the mesenchymal and epithelial markers in breast cancer, which is independent of its coding polypeptide. A series of experiments has proven the limitation of EPR gene expression alterations caused by the translated peptide. Therefore, subsequent studies still focus on the lncRNA itself in spite of the coding function of EPR. Upregulated EPR can induce cancer cell apoptosis through regulating cyclin-dependent kinase inhibitor, epithelial cell transforming growth factor β, and cell cycle arrest (34).

#### Melanoma

The polypeptide MELOE-3 produced by the lncRNA meloe is poorly immunogenic and its translation relies on a cap-dependent mechanism, while two other polypeptides encoded by meloe RNA are less than 50aa in length, with a translation that depends on the IRES, as demonstrated in some melanoma research (53). There is no IRES activity upstream of the MELOE-3-translated ORF, according to *in vitro* translation and transfection experiments. Furthermore, MELOE-3 is expressed in both melanocytes and melanoma cells, but the other two polypeptides are only expressed in melanoma tumor cells. These two polypeptides’ IRES-transactivating factor is specifically activated during the transformation process and that their strong immunogenicity is attributed to their IRES-dependent melanoma specific expression. In brief, these results explain the immune tolerance of the polypeptide MELOE-3 (47).

#### Others

A specific ORF Minion encodes a microprotein and gives rise to myoblast fusion mediated by the transmembrane protein Myomaker. This fusion activity is retained in the human homologue, whose transcript is annotated as lncRNA LOC10192972. This discovery may offer a target for inhibiting the oncolytic fusion of cancer cells (54). LINC00116 is considered as a cervical cancer carcinogen (55), but the overexpression of its coding microprotein Mtln induces the conversion of glycolysis to oxidative phosphorylation in cervical cancer (36). This is distinct from the Warburg effect, which suggests that the metabolism increase in cancer cells is associated with glycolysis instead of oxidative phosphorylation in mitochondria (56). Additionally, the great majority of the lncRNAs covered in our review function in cancer. For instance, LINC01420 can promote the proliferation of nasopharyngeal and pancreatic cancer cells, and it also encodes the polypeptide NoBody (57, 58). In addition, LINC00961 translates the mTORC1 inhibitory peptide and induces the apoptosis of melanoma and oral squamous cell carcinoma (59, 60). However, the cancer functions of their coding products are still unclear and need further research.

### The Function of Long Non-Coding RNA-Encoded Peptides in Other Diseases

Small peptides have become a potential autoimmune particular treatment in the research progress of autoinflammatory disorder. Thus, to find a peptide without off-target stimulation, Niu et al. preferred to focus on human lncRNA MIR155HG which highly expressed in dendritic cell under inflammation, and subsequently proved that miPEP155 (P155) is the MIR155HG-encoded product. P155 can destroy the function of heat shock cognate protein 70 (HSC70), and then regulate antigen transport. Although the P155 sequence was non-existent in mice, results showed the same things as expected, due to the homology HSC70. In addition, imiquimod-induced mouse model research showed that P155 can act on T cells by skewing their polarization states rather than directly monitoring (48). It was reported that the ORF of lncRNA Aw112010 translated an 84aa protein, which generated an innate immune response against infection and inflammation in the inflammatory bowel disease mice models (49). Interestingly, using a pancreatic differentiation system, Bjoern et al. found that lncRNA LINC00261 was necessary for pancreatic endocrine cell development. The lack of LINC00261 will seriously reduce insulin production. Then, they singly mutated seven ORFs in turn and the results showed that neither LINC00261’ ORFs nor their microproteins were involved in the endocrine function of lncRNA itself. Deleting all ORFs simultaneously also would not reduce the stability of LINC00261 transcript (61). It suggested that LINC00261, not its coding peptide, played a practical role in pancreatic endocrine cell development. The same phenomenon also occurs in the lncRNA EPR in breast cancer. These results remind us to identify the function of lncRNA itself or the coding peptide.

### Computational Methods and Data Resources for Predicting Long Non-Coding RNA-Encoded Peptides

In recent years, with the development of high-throughput sequencing, computational methods for predicting lncRNA-encoded proteins have been continuously proposed. In 2009, a ribosome profiling strategy was presented based on the deep sequencing of ribosome-protected mRNA fragments (RPFs) with a length of ~30nt, which could help distinguish “translated RNAs” (62). Wang et al. has reviewed many computational resources for ribosome profiling data processing and interpreting (63). Here, we summarized computational methods through focusing on different key processes of the translation biology based on ribosome profiling data.

#### Challenges for Identifying Ribosome-Protected Messenger RNA Fragments

It is challenging that some RPFs may be mixed with contaminating reads due to technology limitations, such as those derived from ribosomal RNAs (rRNAs). The contaminating reads usually have different length distributions from those real RPFs. This is a key feature to separate bona fide RPFs from contaminating reads such as implemented in FLOSS (64). Rfoot is also designed specifically to distinguish RPFs from other RNA fragments protected by non-ribosome RBPs (65). Meanwhile, true RPFs have shown a three-nucleotide periodicity because of frame preference. For example, a small wavelet transformation method was employed to denoise RPF reads in RiboWave (66). RiboTaper quantifies the significance of periodic ribo-seq reads *via* spectral analysis methods (67).

Identifying the ribosome P-site codon for each read is also a challenge because of the different length distribution of RPFs. Some methods used a fixed offset for reads of different length, such as ORFscore (68). However, the simple handling easily caused erroneous assignment of P-sites. To solve this problem, on the one hand, some tools inferred the P-site positions of RPFs based on the offset of the 5’ ends of RPFs at start or stop codons as well as the consistency of offsets between reads of different lengths such as RiboProfiling and RiboWaltz (69, 70). On the other hand, Scikit-ribo used a random forest classifier considering many features of RPFs including start codons, terminal nucleotides, flanking nucleotides and so on (71).

#### Methods for Identifying “Translated Open Reading Frames”

ORF is usually necessary for translation. In the early stage, computational tool for identifying ORFs only considered start codon and stop codon of genome sequences, such as ORFinder (72). However, some RNAs with ORFs still cannot be translated into proteins or peptides. In order to get a higher accuracy, many tools identified novel ORFs based on more translation features in ribosome profiling data. For example, RiboCode identified canonical and non-canonical ORFs and the associated start codons based on the three-nucleotide periodicity of RPFs (73). RiboHMM used a hidden Markov model (HMM) to identify translated ORFs by leveraging the total abundance and the codon periodicity in RPFs (74). Ribosome profiling with Bayesian predictions (RP-BP) used an unsupervised Bayesian approach to predict translated ORFs through an automatic Bayesian periodic fragment length and ribosome P-site offset Selection (BPPS) (75).

#### Methods for Estimating Translational Efficiency

Translational efficiency (TE) is a key index which estimates translational regulation. Accurate estimation of TE could help predict “translated RNAs.” Most of the methods calculated TE of the ORF as the ratio of reads per kilobase per million mapped reads (RPKM) in ribosome profiling *versus* that in RNA-seq such as RiboProfiling and Plastid (70, 76). However, elongation rate of ribosomes on different ORFs might be different. This would cause errors for estimating TE. To overcome this shortening, Scikit-ribo estimated TE by considering the impact of elongation rate of ribosomes on different codons as well as RNA secondary structures (71). Furthermore, there are some methods focusing on identifying changes in TE under different conditions by modeling the RPF and RNA read counts with some distributions and estimating the significance of TE changes. For example, Xtail used negative binomial distributions (77). Riborex and RiboDiff aimed to run faster by employing a generalized linear model (78, 79).

#### Identification of “Translated RNAs” by MS and Global Translation Initiation Sequencing

MS and global translation initiation sequencing (GTI-seq) have also been incorporated to identify “translated RNAs.” Calviello et al. used ribosome profiling data to make prediction by RiboTaper and used MS spectra to make validation (67). FSPP directly used the overlap of detected small ORF-encoded peptides (SEPs) from ribosome profiling data and MS spectra as target objects (80). GTI-seq could help identify “translated RNAs” by distinguishing ribosome initiation and elongation using two translation inhibitors, lactimidomycin (LTM), and cycloheximide (CHX). Because of combination of ribosome profiling and two different translation-inhibiting chemicals, GTI-seq generates two types of ribosome profiling signal landscapes and improves the identification accuracy of the translation initiation site (81).

#### Databases for Long Non-Coding RNA-Encoded Peptides

Currently, there are several databases containing information about lncRNA-encoded peptides. sORFs.org database provided the coding potential small ORFs identified by ribosome profiling (82). Smprot database recorded small peptides predicted by ribosome profiling data and MS spectra (83). Although numerous ncRNA-encoded peptides have been predicted, however, very few were validated by low-throughput experiment. We also developed a database, ncEP, to collect low-throughput experimentally validated ncRNA-encoded peptides from published papers (84). ncEP also contained an online genome browser showing the genome locations of ncRNAs and proteins or peptides as well as their species conservations. More recently, FuncPEP collected experimentally validated and functionally characterized ncRNA-encoded peptides (85). These resources could enrich the knowledge for translation process. The computational methods and databases for lncRNA-encoded peptides were summarized in **Table 2**.

**Table 2:** Computational methods and databases for long non-coding RNA (lncRNA)-encoded peptides.

Methods or databases	Description	Running environment	URL	Reference
FLOSS	Algorithm for ORF identification based on characteristic read length distribution of true RPFs.	R	NA	(64)
Rfoot	Computational tool for distinguish RPFs from other RNA fragments protected by non-ribosome RBPs and discovering novel non-coding functions of RNAs.	Perl	https://github.com/zhejilab/Rfoot	(65)
RiboWave	Command line tool for denoising RPFs based on wavelet transform and ORF identification and quantification.	R	https://lulab.github.io/Ribowave	(66)
ORFscore	Algorithm for ORF identification based on quantifying the biased distribution of RPFs	R	NA	(68)
RiboTaper	Pipeline for ORF identification based on periodic footprint profiles using the multitaper approach.	R	https://ohlerlab.mdcberlin.de/software/RiboTaper_126/	(67)
RiboWaltz	R package for calculating optimal P-site offsets of RPFs.	R	https://github.com/LabTranslationalArchitectomics/RiboWaltz	(69)
RiboProfiling	R package for transcript quantification and identification of single- or multi-amino acids motifs of RPF accumulation.	R	http://bioconductor.org/packages/RiboProfiling/	(70)
Scikit-ribo	Python package for A-site prediction and robust TE quantification with random forest classifier model.	Python	https://github.com/schatzlab/scikit-ribo	(71)
ORFinder	Web server for identifying ORFs.	Web server	https://www.ncbi.nlm.nih.gov/orffinder/	(72)
RiboCode	Python-based command line tool for *de novo* identification of ORFs based on the three-nucleotide periodicity of RPFs.	Python	https://github.com/xryanglab/RiboCode	(73)
RiboHMM	Hidden Markov model for ORF identification relying on three nucleotide periodicity and coverage of RPFs.	Python	https://github.com/rajanil/riboHMM	(74)
RP-BP	Unsupervised Bayesian pipeline for ORF identification.	Python	https://github.com/dieterich-lab/rp-bp	(75)
Plastid	Python library for nucleotide-resolution analysis of ribosome profiling and TE estimation.	Python	https://github.com/joshuagryphon/plastid	(76)
Xtail	R package for detecting differentially translated ORFs by simulating read counts with negative binomial distribution.	R	https://github.com/xryanglab/xtail	(77)
Riborex	Fast and flexible R package for calculating genome-wide differences in TE using DESeq2 and EdgeR.	R	https://github.com/smithlabcode/riborex	(78)
RiboDiff	Pipeline for differential translation analysis based on generalized linear model of read counts.	Python	https://github.com/ratschlab/RiboDiff	(79)
FSPP	Pipeline for identification of small ORF-encoded peptides (SEPs) by combining ribosome profiling data with MS spectra.	R	https://www.bioinfo.org/FSPP	(80)
sORFs.org	Database for providing the coding potential small ORFs identified by ribosome profiling.	Web server	http://www.sorfs.org	(82)
Smprot	Database for recording small peptides predicted by mass spectrometry (MS) and ribosome profiling data.	Web server	http://bioinfo.ibp.ac.cn/SmProt/	(83)
ncEP	Database for collecting low-throughput experimentally validated ncRNA-encoded peptides from published papers.	Web server	http://www.jianglab.cn/ncEP/	(84)
ncPEP	Database for collecting experimentally validated and functionally characterized ncRNA-encoded peptides.	Web server	https://bioinformatics.mdanderson.org/Supplements/FuncPEP/	(85)

## Discussion

Recent years, more and more studies suggest that some lncRNAs can encode peptides. Most of them are independent of the lncRNA and play important roles in various biological processes. Some lncRNA-encoded peptides may involve in apoptosis inducing and antigen presentation related to autophagy, which implies a close connection with programmed cell death. Furthermore, present coding researches of cancer mainly focus on digestive system, immune system, and skin. Although there is still a vast space to explore, it is no doubt that these peptides will represent new targets for cancer prevention or biomarkers for predicting prognosis of cancer patients.

Here, we described the recent developments of lncRNA-encoded peptides in mammals, including the regulatory pathways, functions, computational predicting methods, and data resources. Since humans have more connections and similarities with other mammals, this review provided more convincing information for researchers focusing on the association between lncRNAs and human diseases. Besides, all lncRNA-encoded peptides with oncogene function are provided as far as we know. Such articles have been published increasingly in recent years and need to be unscrambled systematically. This investigation also carries on the computational methods and data resources for key challenges in the translation process and technology development. The computational methods cover from RPF identification to translated ORF identification and TE estimation. According to the technology development, computational methods are mainly based on ribosome profiling, MS, and GTI-seq. Also, lncRNA encoded peptide-related data resources have been summarized including sORFs.org, Smprot and ncEP databases. Different methods and databases focus on different aspects in the translation process. This summing-up could help researchers to choose the suitable computational methods and databases for their specific interests and give new insights for lncRNA translation.

The progress of identifying coding peptides can refer to the following points. First, bioinformatics technology is used to search for lncRNAs with potential coding ability, especially those related to ribosomes. And it is necessary to mutate each ORF to find the practical one. Second, after proving the generation of the coding peptide, determining endogenously expression or localization is an indispensable step to ensure the peptide is translated from the lncRNA instead of other mRNAs. Then, can the translation be stimulated by eIF4E to enhance or reduce the expression of peptide? After that, it is important to confirm whether lncRNA, its coding peptide, or both have functions. And pull-down assay or immunoprecipitation needs be performed to predict the potential interacting partners combined with MS. Last, consider if the lncRNA with translation should be redefined as a protein coding gene and think about the classification of coding peptide, including micropeptide, polypeptide, and protein.

In the near future, researches about functions of lncRNA-encoded peptides may be a hotpot topic and the coding mechanisms need to be explored in depth. However, there are still some challenges for the further researches. For example, how to predict the coding potential of lncRNA more effectively? Are these lncRNA-encoded peptides different with other peptides? Whether they can be used for diagnosis and prediction of diseases? Whether they can be used as new therapeutic targets or combined with other traditional therapy to improve the curative effects? All of these problems are urgent to be resolved. Interestingly, most of the lncRNA-encoded products in this review are shorter than 100aa. It is difficult to identify long coding proteins with complex biological significance. This may be due to the distribution and the different size of ORFs from lncRNAs and mRNAs. LncRNAs usually harbor ORFs shorter than that of mRNAs. Finally, with the development of new coding prediction tools and genomics technologies, a basis for novel cancer therapeutics should be provided through the exploration of special translation mechanisms and the functions of the coding products.

## Author Contributions

LW and WJ designed the study and drafted the manuscript. JX and HL summarized the theories and drafted the manuscript. All authors contributed to the article and approved the submitted version.

## Funding

This work was supported by grants from the Fundamental Research Funds for the Central Universities (Grant No. 2242020K40131) and the National Natural Science Foundation of China (Grant No. 81972478).

## Conflict of Interest

The authors declare that the research was conducted in the absence of any commercial or financial relationships that could be construed as a potential conflict of interest.
